# An Overview of Bioactive Compounds’ Role in Modulating the Nrf2/Keap1/NF-κB Pathway to Alleviate Lipopolysaccharide-Induced Endometritis

**DOI:** 10.3390/ijms251910319

**Published:** 2024-09-25

**Authors:** Muhammad Zahoor Khan, Wenting Chen, Xiaotong Liu, Xiyan Kou, Adnan Khan, Rahat Ullah Khan, Muhammad Zahoor, Changfa Wang

**Affiliations:** 1 College of Agricultural Science and Engineering, Liaocheng University, Liaocheng 522000, China; 2 Animal Genomics Laboratory, School of Agriculture and Food Science, University College Dublin, D04 V1W8 Dublin, Ireland; 3 CAS Key Laboratory of Pathogenic Microbiology and Immunology, Institute of Microbiology, CAS-TWAS Center of Excellence for Emerging Infectious Diseases, Chinese Academy of Sciences, Beijing 100101, China; 4 Department of Molecular Medicine, Institute of Basic Medical Sciences, University of Oslo, Sognsvannsveien, 90372 Oslo, Norway

**Keywords:** endometritis, Nrf2/Keap1/NF-κB signaling pathway, ROS, oxidative stress, inflammation, bioactive compounds

## Abstract

Endometritis is a common inflammatory condition of the uterine endometrial lining that primarily affects perinatal dairy animals and causes significant economic losses in agriculture. It is usually triggered by pathogenic bacteria and is associated with chronic postpartum reproductive tract infections. Bacterial lipopolysaccharides (LPSs) are known to increase levels of reactive oxygen species (ROS), leading to oxidative stress and inflammation through the activation of the NF-κB signaling pathway and the inhibition of Nrf2 nuclear translocation, which regulates antioxidant response elements (AREs). The effectiveness of the conventional management strategy involving antibiotics is decreasing due to resistance and residual concerns. This review explores the potential therapeutic benefits of targeting the Nrf2/Kelch-like ECH-associated protein 1 (Keap1)/NF-κB signaling pathway to alleviate LPS-induced endometritis. We discuss recent advancements in veterinary medicine that utilize exogenous antioxidants to modulate these pathways, thereby reducing oxidative stress and inflammatory responses in endometrial cells. This review highlights the efficacy of several bioactive compounds that enhance Nrf2 signaling and suppress NF-κB activation, offering protective effects against oxidative damage and inflammation. By examining various in vitro studies, this review emphasizes the emerging role of these signaling pathways in developing new therapeutic strategies that could potentially replace or supplement traditional treatments and mitigate the economic impacts of endometritis in livestock.

## 1. Introduction

Endometritis is the inflammation of the endometrial lining of the uterus. It is frequently associated with chronic postpartum infections of the uterus due to pathogenic bacteria [1,2,3]. This condition is predominantly observed as mucinous or purulent inflammation within the uterine tissues and commonly occurs in perinatal dairy cows [3,4,5]. Reproductive pathologies in livestock present significant challenges to agricultural operations globally, primarily due to their profound adverse impacts on reproductive efficacy, diminished productive capacity, and substantial economic ramifications [6,7,8]. Furthermore, endometritis significantly contributes to economic losses, manifesting through delayed conception, increased frequency of artificial inseminations, escalated veterinary expenditures, elevated incidences of pregnancy losses, infertility, protracted conception rates, and heightened culling rates [9,10]. Additionally, mechanical trauma to the uterus, resulting from artificial insemination, parturition, or obstetric interventions during dystocia, and infections initiated by various pathogenic bacteria represent critical etiological factors for this condition [11]. 

Bacterial lipopolysaccharides (LPSs) are recognized as the primary etiological agent contributing to the development of endometritis, as indicated by recent findings [12,13,14,15]. In cases of LPS-induced endometritis, an elevation in levels of reactive oxygen species (ROS) may precipitate oxidative stress, leading to cellular apoptosis and the regulation of inflammatory responses through the activation of the nuclear factor kappa-light-chain-enhancer of activated B cells (NF-κB) signaling pathway and inhibition of nuclear factor erythroid 2-related factor 2 (Nrf2) translocation to the nucleus [16], which further causes apoptosis and regulates inflammatory changes via the activation of NF-κB signaling and blocking the translocation of Nrf2 to the nucleus [17,18]. 

The clinical management of bovine endometritis has relied predominantly on antibiotic treatments. However, the rising issue of antibiotic resistance and the persistence of drug residues have become significant concerns within the dairy cattle industry [19]. Recent trends in veterinary medicine have seen a shift toward administering bioactive compounds as exogenous antioxidants to alleviate the oxidative stress and inflammatory responses induced by LPS during endometritis, attracting considerable interest [20,21,22]. Furthermore, bioactive compounds are preferred over antibiotics in managing bovine endometritis due to their lower risk of promoting antibiotic resistance and leaving harmful drug residues in animal products. They also provide additional benefits, such as reducing oxidative stress, inflammation, and supporting immune function, rendering them a safer and more sustainable option for animal health.

Several in vitro studies have demonstrated the pivotal role of NF-κB and Nrf2 signaling pathways in mitigating LPS-induced endometritis to relieve oxidative stress and inflammatory changes when supplemented with bioactive compounds [23,24,25,26,27,28]. This review article aims to elucidate the therapeutic potential of targeting the Nrf2/NF-κB signaling pathways in the amelioration of LPS-induced endometritis. It also discusses the efficacy of key bioactive compounds with antioxidant properties that may modulate inflammatory responses and oxidative stress by enhancing Nrf2 signaling and suppressing NF-κB pathway activation, thereby boosting antioxidant and anti-inflammatory responses in endometrial cells to counteract endometritis.

## 2. Materials and Methods

For this comprehensive review, we searched several databases, including PubMed, Scopus, X.MOL, and Web of Science, covering studies published from 2018 to April 2024. The search terms used included “Nrf2 signaling”, “NF-κB signaling”, “endometritis”, “lipopolysaccharide”, “oxidative stress”, “inflammation”, “livestock animals”, “Mouse model”, and “bioactive compounds”. In vitro studies addressing the role of the Nrf2/Kelch-like ECH-associated protein 1 (Nrf2/Keap1) and NF-κB pathways in lipopolysaccharide-induced endometritis were included. Reviews, expert opinions, and meta-analyses were also considered to provide a broad perspective on the topic. The studies included in this review were those that explicitly investigated the Nrf2/Keap1 and/or NF-κB pathways and addressed the mechanisms of action of these pathways in the context of LPS-induced endometritis. Additionally, these studies involved primary animal models or cell lines derived from livestock, specifically dairy cattle. We excluded studies that were not published in peer-reviewed journals or articles published in languages other than English. Book chapters or unpublished data were excluded from this review. Furthermore, we used data from articles only published in 2018–2024. A few studies were also included even up to 2014 merely to keep the clarity of our review.

## 3. LPS-Induced Endometritis

Lipopolysaccharide (LPS), a key component of the outer membrane in gram-negative bacteria, is essential for bacterial survival [29]. When LPS enters the bloodstream, it elicits significant immunological responses. The interaction of LPS with specific cell membrane receptors triggers the release of pro-inflammatory mediators, including tumor necrosis factor-α (TNF-α) and interleukin-1β (IL-1β). This allows researchers to use mammalian models to study inflammatory diseases [30,31,32,33].

Recent studies have shown that prolonged high-concentrate diets in dairy cows can have negative effects, such as increasing LPS concentrations in the circulatory system and inducing a systemic inflammatory response [34]. Elevated LPS levels have been linked to an increase in ROS and a decrease in mitochondrial membrane potential, resulting in mitochondrial dysfunction. This highlights the vulnerability of mitochondrial health to disruption by inflammatory processes [35]. Additionally, LPS exposure has been found to stimulate the upregulation of NADPH Oxidase (NOX), leading to increased ROS production [36]. This rise in ROS not only indicates oxidative stress but also worsens mitochondrial dysfunction, further reducing mitochondrial membrane potential and activating apoptotic pathways [37].

High levels of ROS are significant markers in many inflammatory diseases, with oxidative stress playing a major role in inflammation [38]. LPS-induced oxidative stress can trigger the release of pro-inflammatory cytokines such as IL-1β and TNF-α. These cytokines mediate apoptosis by activating the Fas/FasL-caspase-8/-3 pathway, resulting in increased caspase-3 activity and subsequent cell death [39]. Recent work by Zhao et al. [40] shows that LPS-treated bovine endometrial cells (BEECs) exhibit inflammatory changes, including elevated levels of TNF-α, IL-1β, mitogen-activated protein kinases (MAPK), interleukin-8 (IL-8), increased ROS production, and apoptosis. These changes are accompanied by an increased expression of Bax and caspase-8/-3, as well as a decrease in Bcl2 levels, demonstrating the complex cascade of inflammatory and apoptotic responses triggered by LPS exposure [40]. In contrast, treatment with cecropin A, a naturally occurring antimicrobial peptide, significantly mitigates these responses. Cecropin A reduces TNF-α, IL-1β, and IL-8 levels and inhibits the MAPK pathway, indicating its potent anti-inflammatory properties [40]. It also lowers ROS levels, thereby alleviating LPS-induced oxidative stress and mitochondrial dysfunction. This effect is achieved through the downregulation of NOX and the upregulation of catalase (CAT), glutathione peroxidase (GPX), and superoxide dismutase (SOD), highlighting its antioxidant capabilities. Furthermore, cecropin A inhibits apoptosis, particularly by suppressing the mitochondrial-dependent apoptotic pathway, as evidenced by the Fas/FasL-caspase-8/-3 cascade. The increased Bcl-2/Bax ratio, an important regulator of apoptosis, demonstrates the protective role of cecropin A against cell death [40]. Consistent with those findings, a study has shown that LPS-induced endometritis is associated with elevated inflammatory changes, including the upregulation of the *C-C motif chemokine Ligand 2 (CCL2)*, *interleukin-6 (IL-6)*, and *NF-κB* [41]. 

A study by El-Sayed et al. [42] reported a significant upregulation of toll-like receptor 4 (TLR4), IL-8, IL-17, NF-κB, and Keap1, along with a downregulation of key antioxidant enzymes such as SOD and CAT, as well as Nrf2, in buffalo suffering from endometritis. The upregulation of these inflammatory markers highlights the heightened immune response and oxidative stress associated with the condition. TLR4 plays a critical role in recognizing pathogens and activating downstream inflammatory pathways, including the release of IL-8 and IL-17, which are key mediators in the inflammatory response. The activation of NF-KB further amplifies this response, promoting the transcription of pro-inflammatory cytokines and perpetuating inflammation in the endometrial tissue. In addition, the downregulation of Nrf2, a key regulator of the antioxidant defense system, along with the reduced expression of SOD and CAT, suggests a weakened antioxidant response in the endometriotic buffalo [42]. The findings of El-Sayed et al. [42] are consistent with previous research by Kharayat et al. [43], which also reported a higher expression of TLRs in buffalo with endometritis. The upregulation of TLRs, particularly TLR4, underscores their central role in sensing microbial infections and initiating inflammatory responses. In summary, the modulation of apoptosis and inflammation by cecropin A in LPS-treated BEECs highlights its dual therapeutic capabilities, provides insights into its mechanisms of action, and suggests potential applications in treating endometrial inflammatory conditions.

## 4. Bioactive Compounds Targeting Nrf2/Keap1 Signaling for Mitigating LPS-Induced Endometritis

The signaling pathway of Nrf2 plays a critical role in protecting endometrial cells against oxidative stress and inflammation. This is particularly relevant in diseases like endometritis, which is often caused by bacterial infections, especially those initiated by LPS from gram-negative bacteria. Normally, Nrf2 is held in the cytoplasm by a protein called Keap1, which also targets it for degradation through ubiquitination and subsequent proteasomal degradation. However, under oxidative stress, such as that induced by LPS in endometritis, Nrf2 dissociates from Keap1, moves into the nucleus, and triggers the transcription of genes under the antioxidant response element (ARE) promoter. These genes encode essential antioxidant enzymes and detoxifying proteins, crucial for counteracting oxidative stress and maintaining cellular redox homeostasis. 

**Figure 1:**
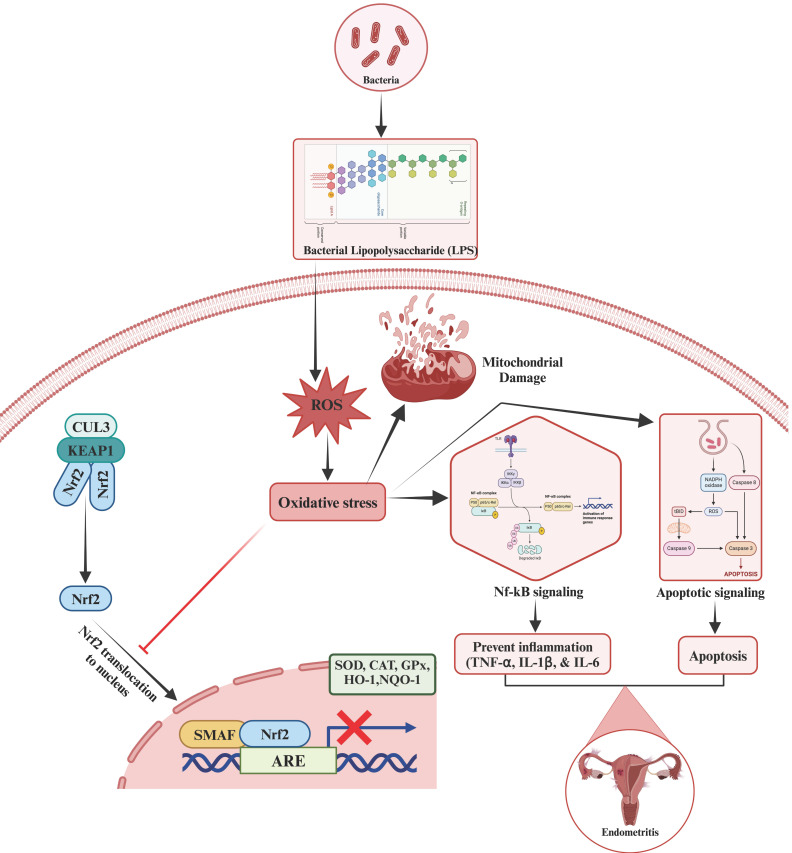
The mechanism of Lipopolysaccharides (LPS)-induced endometritis via elevated levels of oxidative stress followed by the activation of the nuclear factor-κB (NF-κB) signaling pathway, which further blocks the activation of NF-E2-related factor-2 (Nrf2) by inhibiting the degradation of Kelch-like ECH-associated protein 1 (Keap1).

Additionally, NF-κB, a key transcription factor, regulates the expression of the genes involved in inflammatory responses. In conditions like LPS-induced oxidative stress, the NF-κB pathway becomes activated, leading to the production of pro-inflammatory cytokines, chemokines, and adhesion molecules. Nrf2, by increasing the levels of antioxidant proteins, reduces the concentrations of ROS and nitric oxide (NO), thereby attenuating NF-κB activation and subsequent inflammatory responses (Figure 1). Elevated levels of ROS, a consequence of LPS exposure, worsen cellular damage and further stimulate inflammatory pathways, including NF-κB. Nrf2, on the other hand, counteracts these effects by enhancing the cellular antioxidant capacity. This is evident from the upregulation of enzymes such as NAD(P)H quinone dehydrogenase 1 (NQO1), heme oxygenase-1 (HO1), SOD, CAT, and GPX, which collectively reduce oxidative stress and limit cellular damage.

Recent studies have shed light on the protective role of bioactive compounds in this context (Table 1). Gao et al. [44] demonstrated that the pre-administration of luteolin in mice mitigated severe uterine tissue damage and inflammation induced by *Staphylococcus aureus.* This was accompanied by a dose-dependent reduction in myeloperoxidase (MPO) activity, as well as in levels of TNF-α, IL-1β, and IL-6. Luteolin also preserved the integrity of the endometrial barrier, inhibited NF-κB activation, and enhanced the expression of Nrf2 and HO-1. The knockdown of Nrf2 reversed these protective effects, highlighting its crucial role in modulating the Nrf2 signaling pathway during *S. aureus*-induced endometritis. Additionally, Zhao et al. [45] found that citral effectively mitigated LPS-induced endometritis and inhibited ferroptosis by decreasing levels of malondialdehyde (MDA) and iron (Fe2+) while increasing levels of adenosine triphosphate (ATP) and glutathione (GSH). Citral also promoted the upregulation of Nrf2 and HO-1 expression and reduced NF-κB activation. In Nrf2-knockdown mice, the absence of Nrf2 significantly diminished the protective effects of citral against ferroptosis and endometritis, confirming that citral’s protective mechanism in LPS-induced endometritis operates through the modulation of the Nrf2 signaling pathway [45].

Similarly, a study by Jin et al. [46] investigated the impact of liquiritin on lipopolysaccharide-induced human endometrial epithelial cells. They found that liquiritin significantly decreased cell viability, inflammatory changes (downregulation of interleukin-1β, tumor necrosis factor alpha, and interleukin-6), and apoptosis through the activation of the Keap1/Nrf2/HO-1 signaling pathway, which reduced oxidative stress and inflammatory responses via its downstream genes (Keap1, Nrf2, NQO1, and HO-1) [46]. The use of ML385, an inhibitor of the Nrf2 pathway, reversed the beneficial effects of liquiritin and confirmed the critical role of the Keap1/Nrf2/HO-1 pathway in mitigating inflammation and oxidative stress [46]. Consistently, another study reported that oxycodone alleviated human endometrial stromal cell injury caused by mifepristone [47]. They observed higher expressions of key antioxidant response-linked biomarkers such as Keap1, Nrf2, HO-1, and NQO1 and the suppression of the level of caspase3 in response to oxycodone for the prevention of endometritis [47]. Furthermore, a study by Song P et al. [48] found that LPS exposure led to the upregulation of Nrf2 expression, concurrent with endoplasmic reticulum stress (ERS) in BEECs. Increased Nrf2 activation correlated with heightened transcriptional activity of pro-inflammatory markers, including TNF-α, the p65 subunit of the NF-κB, IL-6, and IL-8 within BEECs [48]. Furthermore, Nrf2 activation coincided with ERS induction. Conversely, Nrf2 depletion attenuated the expression levels of TNF-α, p65, IL-6, and IL-8. Notably, Nrf2 silencing corresponded with diminished expression of ERS-associated genes encoding glucose-regulated protein 78 (GRP78), protein kinase RNA-like endoplasmic reticulum kinase (PERK), eukaryotic initiation factor 2 alpha (eIF2α), activating transcription factor 4 (ATF4), and C/EBP homologous protein (CHOP) within BEECs [48]. These observations underscore the activation of Nrf2 and ERS during the inflammatory cascade within BEECs. Additionally, Nrf2 facilitates the inflammatory milieu by instigating the protein kinase RNA-like ER kinase (PERK) signaling pathway within the context of ERS, thereby precipitating apoptosis in BEECs. In line with this, a recent study found that Nrf2 was enhanced by Berberine to relieve LPS-endometritis and apoptosis in BEECs [49,50]. Berberine ameliorated inflammatory changes via the downregulation of IL-1β, IL-6, and TNF-α and enhanced the antioxidant response via elevated levels of Nrf2 and HO-1 [49,50]. The prevention of endometritis in BEECs was attributed to the Keap1/Nrf2 signaling pathway [49,50]. Similarly, another study reported that curcumin supplementation relieved endometritis caused by Bisphenol AF in caprine endometrial epithelial cells (EECs) [51]. The supplementation suppressed the levels of reactive oxygen species, inflammation, and apoptosis. Additionally, acai berry supplementation in rats exhibited effectiveness against endometritis and ameliorated oxidative stress and apoptosis by enhancing autophagy. This was followed by elevated expressions of Nrf2, HO-1, and NQO1 in the nucleus [52]. Furthermore, the study observed the promoting activity of the autophagy-related 1(ATG1)/Unc-51 like kinase-1(ULK1) complex and Bcl2 in the EECs of rats.

**Figure 2:**
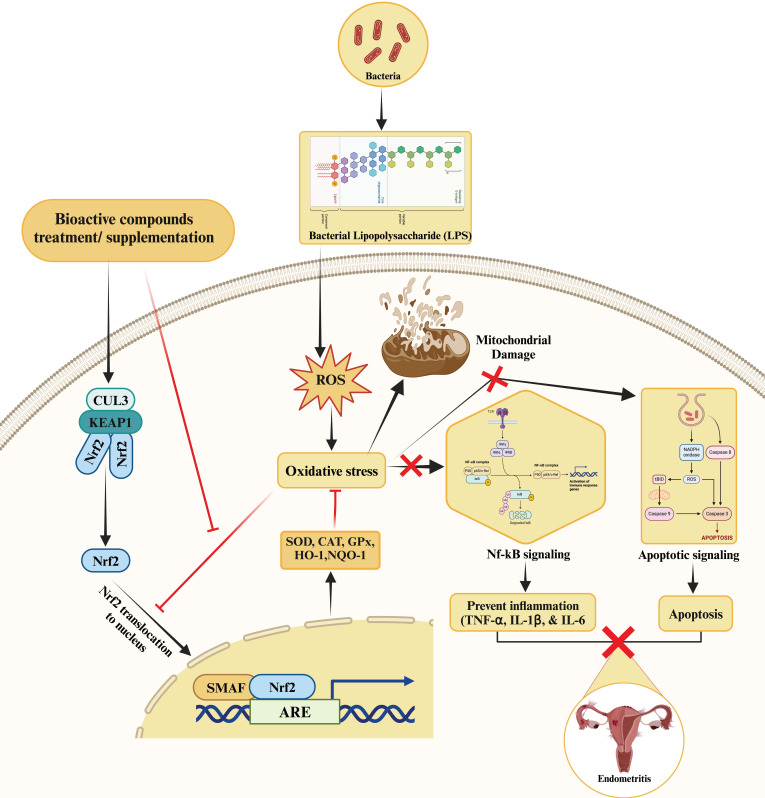
The molecular mechanism by which bioactive compounds mitigate LPS-induced endometritis. The bioactive compounds activate the Nrf2/Keap1 signaling pathway, which in turn modulates NF-κB activity. Nrf2, when released from its inhibitor Keap1, translocates to the nucleus, leading to the expression of antioxidant and anti-inflammatory genes. Simultaneously, the activation of Nrf2 attenuates the pro-inflammatory NF-κB signaling, reducing inflammatory responses. Collectively, these pathways help to counteract the inflammatory damage induced by LPS in endometrial tissue.

Crocetin, a major active constituent from saffron extract, also showed protective effects against reactive oxygen species, inflammation, and apoptosis [53]. In rats, the administration of crocetin relieved inflammation and apoptosis and decreased oxidative stress. This was achieved through the upregulation of Nrf2, heme oxygenase (HO)-1, and NQO1, as well as decreased MDA content [53]. It was concluded that crocetin administration is used to treat endometritis via the Nrf2/HO-1 signaling pathway in the endometrial cells of rats. Icariin was found to alleviate *Escherichia coli* lipopolysaccharide-mediated endometritis in mice by inhibiting inflammation and oxidative stress. A study revealed that *Trueperella pyogenes* exotoxin induced oxidative stress and inflammatory changes by significantly downregulating the level of Nrf2 and its downstream genes HO-1 and NQO-1 while increasing the expressions of NF-κB, IL-1β, IL-6, and TNF-α [54]. Treatment with chlorogenic acid improved the levels of Nrf2, HO1, and NQO1 and reversed the inflammatory changes to prevent endometritis in *Trueperella pyogenes* exotoxin-treated mouse endometrial epithelial cells [54]. The study concluded that chlorogenic acid relieved *Trueperella pyogenes* exotoxin-induced endometritis in mice through the utilization of Nrf2/HO1/NF-κB signaling pathways. In addition, andrographolide [55] and tanshinone IIA [56] upregulated the expressions of antioxidant response genes and downregulated MDA levels to relieve oxidative stress and inflammatory changes induced by LPS in BEECs. These drugs prevented LPS-induced endometritis through the activated Nrf2 signaling pathway [55,56]. Oxygen and glucose deprivation (OGD)-reoxygenation (OGDR) induced oxidative injury to endometrial cells, leading to increased levels of reactive oxygen species production, lipid peroxidation, and mitochondrial depolarization, as well as reduced cell viability and necrosis [57]. Ginsenoside Rh3 attenuated endometrial cell injury via regulated Nrf2 signaling, resulting in the upregulation of antioxidant-related genes (*HO1*, *NQO1*, and glutamate-cysteine ligase (*GCLC*)) [57]. Similarly, sulforaphane alleviated endometriosis in rats by decreasing pro-inflammatory cytokines such as *IL6*, *IL-1β*, and *TNF-α* and suppressing levels of DOX2 and INOS while also upregulating *Keap1* and *Nrf2* [58]. Figure 2 illustrates the molecular mechanisms by which bioactive compounds alleviate endometritis.

**Table 1:** Role of bioactive compounds in targeting Nrf2 signaling for mitigating LPS-induced endometritis.

Therapeutic Agent	Target Pathway	Outcomes	Species	References
Liquiritin	Keap1/Nrf2/HO-1 signaling pathway	✧ Alleviated LPS-induced oxidative stress. ✧ Improved mice mammary epithelial cell viability and antioxidant capacity. ✧ Restored mammary health by enhancing Nrf2, T-AOC, and MAPK expression.	HEECs	[46]
Meloxicam	Nrf2/NF-κB pathways	✧ Reduced the expression of NF-κB, MyD88 followed by suppression of pro-inflammatory cytokines. ✧ Relieved oxidative stress by enhancing Nrf2 expression.	BEECs	[59]
Chicoric acid	Nrf2/HO-1 signaling pathway	✧ Prevented inflammatory changes and endometritis via inhibition of NF-κB and elevated levels of Nrf2 and HO-1.	Mouse endometrial epithelial cells (MEECs)	[60]
Icariin	Nrf2/NF-κB signaling pathways	✧ Inhibited the production of TLR4, NF-κB, IL-1ß, IL-6, and TNF-α) and enhanced the production of anti-inflammatory cytokines (IL-10) to ameliorate inflammatory changes. ✧ In addition, icariin suppressed the levels of MDA and ROS and improved Nrf2, NQO1, HO-1, SOD1, CAT, and GPX expressions to relieve the oxidative stress induced by LPS. ✧ LPS-induced endometritis was ameliorated via Nrf2/NF-κB signaling pathways.	MEECs	[61]
Platelet-rich plasma	Nrf2/HO-1 signaling pathway	✧ Platelet-rich plasma infusion relieved LPS endometritis, enhanced endometrial cell viability, decreased apoptosis, and diminished inflammatory changes via activated Nrf2/HO-1 signaling pathway.	MEECs	[62]
Polydatin	NF-κB/Nrf2 signaling pathway	✧ Pro-inflammatory activator (NF-κB, IL-1 β, TNF-α) levels were suppressed. ✧ Oxidative stress was alleviated via the upregulation of Nrf-2. ✧ Relieved endometritis by activating the NF-κB/Nrf2 signaling pathway.	HEECs	[63]
Piceatannol	NF-κB//Nrf2/HO-1 signaling pathway	✧ Piceatannol supplementation prevented estradiol-induced endometrial hyperplasia in rats by the activation of NF-κB/Nrf2/HO-1 signaling pathways. ✧ Reduced the level of NF-κB, IL-1ß, IL-6, and TNF-α. ✧ Enhanced the antioxidant response via Nrf2 and HO-1 expressions.	Rat EECs	[64]
Aucubin	Keap1/Nrf2/NF-κB signaling pathway	✧ Aucubin-ameliorated inflammatory changes reduced the expressions of NF-κB p65, IκB phosphorylation, TNF-α, IL-1β, IL-6, COX-2, and iNOS. ✧ Increased the mRNA levels of Keap1, Nrf2, HO-1, and NQO1 to promote antioxidant response. ✧ The curative effect of aucubin on endometritis was due to activated NF-κB/Keap1/Nrf2 signaling pathways.	BEECs	[65]
Methyl ester of 2-cyano-3,12-dioxooleana-1,9-dien-28-oic acid	Nrf2/NF-κB signaling pathways	✧ Promoted Nrf2, NQO-1, HO-1, GSH, and SOD1 expressions. ✧ Inhibited inflammatory changes via decreased expression of pro-inflammatory cytokines (NF-κB, TNF-α, IL-1β, IL-6, and COX-2).	Rat EECs	[66]
Dimethyl itaconate	TLR4/NF-κB/Nrf2/HO-1 signaling pathway	✧ LPS by binding with TLR4 induced pro-inflammatory changes (elevated levels of TNF-α, IL-1β, IL-6), increased uterine MPO activity, and activated NF-κB in MEECs. ✧ Decreased the expressions of Nrf2 and HO-1. ✧ Dimethyl itaconate ameliorated LPS-induced endometritis via the TLR4/NF-κB/Nrf2/HO-1 signaling pathway followed by suppressed levels of pro-inflammatory cytokines and an enhancement of the antioxidant response (Elevated levels of Nrf2 and HO-1).	MEECs	[67]
Hydroxytyrosol	Nrf2 signaling pathway	✧ Relieved the endometritis in BEECs by enhancing the antioxidant response (Nrf2, HO-1, and NGO1) and the downregulation of pro-inflammatory cytokines (TNF-α and IL-6).	BEECs	[68]
Apigenin	NF-κB/Nrf2/HO-1 signaling pathway	✧ Prevented the endometritis via inhibition of MPO, NF-Κb, TNF-α, and IL-1β. ✧ Enhanced the expressions of Nrf2 and HO-1 levels and suppressed the MDA content.	MEECs	[69]

Bovine endometrial epithelial cells (BEECs); Human endometrial epithelial cells (HEECs); mouse endometrial epithelial cells (MEECs).

## 5. Bioactive Compounds Targeting NF-κB Signaling for Alleviating LPS-Induced Endometritis

Recent studies have extensively discussed the potential role of NF-κB signaling as a therapeutic target for endometrial diseases in humans and animals [70,71,72,73]. In a notable study by Jiang and Xu [74], it was demonstrated that Timosaponin AIII, an active pharmacological component of *A. asphodeloides*, plays a therapeutic role in combating *E. coli*-induced endometritis by inhibiting inflammatory responses and oxidative stress. This is achieved through the downregulation of NF-κB and TLR4 in mouse endometrial cells. Similarly, Liu F et al. [75] highlighted that Timosaponin enhances antioxidant responses by positively regulating the Nrf2/HO-1 signaling pathway. Further studies corroborate these findings. For instance, Jiang K et al. [76] used a mouse model and BEECs to show that Fisetin ameliorates endometritis by inhibiting TLR4 expression and the nuclear translocation of NF-κB, thereby reducing the secretion of pro-inflammatory mediators (TNF-α, IL-1β, and IL6). Furthermore, the study found that fisetin alleviates oxidative stress in BEECs and MEECs by upregulating Nrf2 and HO-1 levels. This study underscored the potential of targeting the Nrf2/HO-1/TLR4-NF-κB signaling pathways with Fisetin for the treatment of LPS-induced metritis. The interaction between NF-κB and Nrf2 signaling was further explored in LPS-induced endometritis in goat mammary epithelial cells (GMECs) [77]. The study found that Nrf2 combines with p25 to inhibit the production of pro-inflammatory cytokines (TNF-α, IL-1β, IL-6, or IL-8). Conversely, blocking Nrf2 in GMECs significantly exacerbated inflammatory changes and oxidative stress by activating the TLR4/NF-κB pathway. In a similar way, Yang Y et al. [78] reported that Neisseria gonorrhoeae activates NF-κB/p65 and suppresses Nrf2 levels via TLR2/TLR4, inducing endometritis in human females. However, the knockdown of TLR2/TLR4 significantly reduced inflammatory responses, downregulated NF-κB/p65, and elevated Nrf2 levels, thereby ameliorating endometritis.

Furthermore, Wang D et al. [79] observed an increased expression of latent transforming growth factor-beta binding protein 2 (LTBP2) in the serum of females with endometriosis, which promotes inflammation through the phosphorylation of the NF-κB signaling pathway, facilitating disease progression. On the other hand, it was reported that the peroxisome proliferator-activated receptor γ (PPARγ) inhibits pro-inflammatory cytokines (TNFα, IL-1β, and IL-6), TLR4, and NF-κB, relieving LPS-induced endometritis in pig endometrial epithelial cells (EECs) [80]. The role of hydroxysafflor yellow A (HYA) in mitigating mouse endometrial inflammation induced by *S. aureus* was documented by He S et al. [81]. HYA suppressed TLR2, resulting in reduced expressions of TNF-α, IL-1β, and IL-6. This inhibition of the TLR2/NF-κB pathway prevented endometrial inflammation. Similar studies by Qin X et al. [82] and Liu J et al. [83] identified TNFAIP3 interacting protein 2 (TNIP2) and miR-488 as key factors in blocking the expression of NF-κB and its downstream genes (IL-6, IL-1β, and TNF-α) to prevent LPS-induced endometritis. Consistently, another study revealed that autophagy was suppressed in LPS-induced endometritis in BEECs, leading to the upregulation of NF-κB, IL-6, IL-8, and TNF-α 1 [84]. Additionally, selenomethionine was reported to alleviate endometritis caused by *Escherichia coli* (*E. coli*) by upregulating ZO-1 and occludin and inhibiting NF-κB signaling [85]. Interestingly, supplementation with *Clostridium butyricum* was found to alleviate inflammatory changes and prevent endometritis in MEECs. This was achieved by inhibiting the myeloperoxidase (MPO)- and TLR4-mediated phosphorylation of the NF-κB signaling pathway and the activity of histone deacetylase (HDAC) [86]. Furthermore, decreased levels of MDA and increased expression of the tight junction proteins (TJPs) ZO-1, claudin-3, and occludin were observed in MEECs in response to *C. butyricum* [86], suggesting that *C. butyricum* significantly attenuates inflammatory changes and relieves endometritis by inhibiting the NF-κB signaling pathway. Moreover, a study revealed that interferon-tau (IFN-τ) prevents *S. aureus*-induced endometritis by downregulating the expressions of NF-κB, matrix metalloproteinase 9 (MMP 9), TLR2, TNF-α, IL-1β, and IL-6 levels [19]. 

A study examined the immunomodulatory effects of curcumin on inflammatory responses in bubaline endometrial stromal cells induced by LPS [87]. Furthermore, they found that LPS significantly increased PGE2 and pro-inflammatory cytokines (IL-1β, TLR4, IL-6, IL-8, and TNF α), while curcumin inhibited these effects [87,88]. A recent study demonstrated that Lactococcus lactis, particularly in its nano-encapsulated form, serves as a promising alternative to conventional antibiotic treatments for clinical endometritis in buffaloes. In this study, four intrauterine injections, each containing 10⁹ CFU of L. lactis in 50 μL of saline buffer, were administered on alternate days (days 1, 3, 5, and 7) [89]. The treatment significantly reduced uterine inflammation, pathogen load, and systemic inflammation while enhancing reproductive outcomes. Moreover, the study observed a notable decrease in serum non-esterified fatty acids (NEFA), polymorphonuclear cells (PMN), and purulent vaginal discharge (PVD), alongside the downregulation of endometrial pro-inflammatory mRNA expressions of *tumor necrosis factor alpha-induced protein (TNFAIP7)*, *interleukin-6 (IL6)*, and *IL-1β* [90]. Another study examined the effect of the intrauterine infusion of proteolytic enzyme on reducing endometrial inflammation and improving reproductive performance in buffaloes with subclinical endometritis [91,92]. Their results showed that the proteolytic enzyme treatment significantly reduced uterine inflammation markers, including PMN levels and cytokines like IL-1β and TNF-α, both in the uterus and in serum. Acute-phase proteins like amyloid-A and haptoglobin also decreased post-treatment in response to proteolytic enzyme treatment [90,91]. For ease of reference, Table 2 provides a summary of studies targeting bioactive compounds that aim to mitigate LPS-induced endometritis by modulating the NF-κB signaling pathway.

## 6. Conclusions

In conclusion, this review provides a comprehensive understanding of the critical roles played by the Nrf2/Keap1/NF-κB signaling pathways in mediating inflammatory and oxidative responses in LPS-induced endometritis in vitro. We emphasize the importance of these pathways in regulating the cellular mechanisms underlying inflammation and oxidative stress in endometrial cells. The interplay between Nrf2 and NF-κB signaling is particularly significant, as Nrf2 enhances antioxidant defenses to counteract the pro-inflammatory actions mediated by NF-κB in the presence of LPS. This delicate balance between mitigating oxidative stress and modulating the inflammatory response presents a promising target for therapeutic interventions. Recent studies have highlighted the potential of bioactive compounds that can modulate these pathways, offering innovative strategies for managing endometritis in vitro. Future research should focus on conducting detailed mechanistic studies and clinical trials to validate the efficacy of these compounds in endometritis treatment. Overall, based on the existing, available literature, we suggest that targeting the Nrf2/Keap1/NF-κB signaling pathways via bioactive compounds holds great promise for improving reproductive health and can be considered a potential therapeutic target to mitigate endometritis in livestock animals and humans.

## Figures and Tables

**Table 2:** Role of bioactive compounds in targeting NF-κB signaling for mitigating LPS-induced endometritis.

Therapeutic Agent	Target Pathway	Outcomes	Species	References
Rosiglitazone	TLR4/NF-κB signaling pathway	✧ Suppressed the levels of IL-1β, NF-κB, IL-6, and TLR4. ✧ Alleviated endometritis via the inhibition of the TLR4/NF-κB signaling pathway.	MEECs	[12]
Platelet-rich plasma	TLR4/NF-κB signaling pathway	✧ Ameliorated inflammatory changes in mouse endometrial cells via the suppression of pro-inflammatory cytokines (TNF-α, IL-1β, and IL-6). ✧ The TLR4 and NF-κB were blocked to relieve endometritis in MEECs.	MEECs	[13]
Nuciferine	MyD88/NF-κB signaling pathway	✧ Prevented LPS-induced endometritis by inhibiting NF-κB, MAPK, and MYD88 expressions.	MEECs	[92]
Flavonoids isolated from *Clinopodium chinense*	TLR4/NF-κB/NLRP3 signaling pathway	✧ An in vitro experiment revealed that flavonoid administration relieved LPS-induced endometritis by inhibiting levels of *NLR family pyrin domain-containing 3 (NLRP3)*, *TLR4*, *IL-18*, *IL-1β*, and *TNF-α*. ✧ LPS-induced endometritis was prevented by suppressing the activation of the TLR4/NF-κB/NLRP3 signaling pathway.	MEECs	[93]
Kynurenic acid	GRP35/NF-κB signaling pathway	✧ LPS-induced endometritis was prevented by kynurenic acid via reduced secretion of *IL-6*, *TNF-α*, and *IL-1β*. ✧ Furthermore, the NF-κB was blocked and upregulated the level of *G protein-coupled receptor 35 (GPR35)*. ✧ The tight junction proteins occludin and ZO-1 levels were enhanced. ✧ Overall, kynurenic acid administration protected against LPS-induced endometritis by maintaining epithelial barrier permeability and suppressing pro-inflammatory responses and inhibited uterine pathological injury and neutrophil infiltration.	MEECs	[94]
Rhizomes of *Cyperus rotundus* (Traditional medicine)	Akt/NF-κB signaling pathway	✧ Alleviated endometritis via inhibition of Akt and NF-κB pathway activation in human endometrial cells.	HEECs	[95]
Sodium butyrate	NF-κB signaling pathway	✧ Reduced LPS-induced inflammatory cytokines TNF-α and IL-1β. ✧ Blocked the activation of NF-κB signaling to relieve the endometritis.	MEECs	[96]
Leonurine	NF-κB signaling pathway	✧ Inhibited the expression levels of LPS-induced IL-6, TNF-α, and IL-1β in uterus tissues and BEECs. ✧ The activation of TLR4 and NF-κB was blocked. ✧ The LPS-induced endometritis was prevented by leonurine.	BEECs	[97]
PGE2	TLR4-NF-κB signaling pathway	✧ Downregulated the expressions of COX2, TLR4, IL-8, NF-Κb, and IL-6 to reverse the LPS-induced inflammatory changes and, alternatively, the endometritis BEECs.	BEECs	[98]
Thymol	NF-κB signaling pathways	✧ Attenuated LPS-induced endometritis in mice by downregulating *MPO*, *TNF-α*, and *IL-1β*. ✧ Inhibited the expression of *TLR4* and the activation of NF-κB signaling and consequent inflammatory changes.	MEECs	[99]

## Data Availability

All the data are available in the manuscript.
